# Comparison of Astigmatic Correction after Femtosecond Lenticule Extraction and Small-Incision Lenticule Extraction for Myopic Astigmatism

**DOI:** 10.1371/journal.pone.0123408

**Published:** 2015-04-07

**Authors:** Hidenaga Kobashi, Kazutaka Kamiya, Mohamed A. Ali, Akihito Igarashi, Mohamed Ehab M. Elewa, Kimiya Shimizu

**Affiliations:** 1 Department of Ophthalmology, University of Kitasato School of Medicine, Kanagawa, Japan; 2 Department of Ophthalmology, Faculty of Medicine, Minia University, Minia, Egypt; Medical College of Soochow University, CHINA

## Abstract

**Purpose:**

To compare postoperative astigmatic correction between femtosecond lenticule extraction (FLEx) and small-incision lenticule extraction (SMILE) in eyes with myopic astigmatism.

**Methods:**

We examined 26 eyes of 26 patients undergoing FLEx and 26 eyes of 26 patients undergoing SMILE to correct myopic astigmatism (manifest astigmatism of 1 diopter (D) or more). Visual acuity, cylindrical refraction, the predictability of the astigmatic correction, and the astigmatic vector components using Alpin’s method, were compared between the two groups 3 months postoperatively.

**Results:**

We found no statistically significant difference in manifest cylindrical refraction (p=0.74) or in the percentage of eyes within ± 0.50 D of their refraction (p=0.47) after the two surgical procedures. Moreover, no statistically significant difference was detected between the groups in astigmatic vector components, namely, surgically induced astigmatism (0.80), target induced astigmatism (p=0.87), astigmatic correction index (p=0.77), angle of error (p=0.24), difference vector (p=0.76), index of success (p=0.91), flattening effect (p=0.79), and flattening index (p=0.84).

**Conclusions:**

Both FLEx and SMILE procedures are essentially equivalent in correcting myopic astigmatism using vector analysis, suggesting that the lifting or non-lifting of the flap does not significantly affect astigmatic outcomes after these surgical procedures.

## Introduction

The femtosecond laser is one of the most revolutionary inventions in recent medical technology that has been used mainly in ophthalmology for laser in situ keratomileusis (LASIK). It is employed as an alternative to the mechanical microkeratome for precisely and reproducibly creating corneal flaps. A recent breakthrough in this technology is refractive lenticule extraction (ReLEx), which requires neither a microkeratome nor an excimer laser, but uses only the femtosecond laser system as an all-in-one device for flap and lenticule processing. The first clinical results were obtained in highly myopic eyes [1], and in blind or amblyopic eyes [2]. Additionally, the ReLEx technique, which can be used for femtosecond lenticule extraction (FLEx) by the raising of the flap, or by small-incision lenticule extraction (SMILE) (without flap raising), has been proposed as an alternative to conventional LASIK for the correction of refractive errors [3–8]. SMILE is theoretically equivalent to FLEx for the surgical techniques except for the flap raising. In the prospective, randomized, intraindividual comparative study, it was found that both FLEx and SMILE were beneficial in all measures of safety, efficacy, predictability, and stability for the correction of myopia throughout the 6-month follow-up [9]. As for the correction of myopic astigmatism, refractive surgeons may be concerned about the difference in astigmatic correction between FLEx and SMILE. However, to our knowledge, no comparison of the equivalent astigmatic correction after FLEx and SMILE has so far been conducted. The current study was designed to compare the astigmatic correction between FLEx and SMILE in eyes with myopic astigmatism.

## Patients and Methods

Twenty-six eyes of 26 consecutive patients (10 men and 16 women) who underwent FLEx and 26 eyes of 26 consecutive patients (9 men and 17 women) who underwent SMILE, for the correction of myopic astigmatism were included in this experimental study. One eye from each patient was chosen randomly for the measurement. Some of the subjects were those in our preceding report on visual and refractive outcomes after FLEx and SMILE [9]. Otherwise, we performed FLEx up to and including November 2011, and SMILE from December 2011 onwards, regardless of the amount of preoperative manifest equivalent refraction or cylindrical refraction. The sample size in this study offered 94% statistical power at the 5% level in order to detect a 0.10-D difference in manifest cylinder, when the standard deviation (SD) of the mean difference was 0.10 D [10]. The inclusion criteria for this study were as follows: unsatisfactory correction with spectacles or contact lenses, manifest spherical equivalent of -1 to -9 D, manifest cylinder of -1.00 to -2.75 D, sufficient corneal thickness (an estimated total corneal thickness of >400 μm and an estimated residual thickness of the stromal bed of >250 μm), endothelial cell density ≥1800 cells/mm^2^, and no history of ocular surgery, severe dry eye, progressive corneal degeneration, cataract or uveitis. Eyes with keratoconus were excluded from the study by using the keratoconus screening test that employs Placido disk videokeratography (TMS-2, Tomey, Nagoya, Japan). Written informed consent was obtained from all patients after explanation of the nature and possible consequences of the study. The study was approved by the Institutional Review Board of Kitasato University and followed the tenets of the Declaration of Helsinki. The author’s Institutional Review Board waived the requirement for informed consent for this retrospective study.

### FLEx and SMILE surgical procedures

Both FLEx and SMILE were performed under topical anesthesia (0.4% oxybuprocaine, Benoxyl; Santen, Osaka, Japan) using the VisuMax femtosecond laser system (Carl Zeiss Meditec AG, Jena, Germany) with a 500 kHz repetition rate. The laser was visually centered on the pupil. A small curved interface cone was used in all cases. The main refractive and non-refractive femtosecond incisions were performed in the following automated sequence: the posterior surface of the lenticule (spiral-in pattern), then the anterior surface of the lenticule (spiral-out pattern), followed by a side cut of the flap. The femtosecond laser parameters were as follows: 120 μm flap thickness, 7.5 mm flap diameter, 6.5 mm lenticule diameter, 140 nJ power for lenticule and flap, and a 310-degree side cut (superior hinge) with side cut angles of 90 degrees for FLEx; and 120 μm cap thickness, 7.5 mm cap diameter, 6.5 mm lenticule diameter, 140 nJ power for lenticule and cap and a 50-degree side cut for access to the lenticule with angles of 90 degrees for SMILE. After the suction was released, the patient was moved toward the observation position under the VisuMax integrated surgical microscope. For FLEx, after completion of the laser sequence, a Siebel spatula was inserted under the flap near the hinge and the flap was lifted; and the refractive lenticule was next grasped with forceps and extracted. The flap was then repositioned. For SMILE, a thin spatula was inserted through the side cut over the roof of the refractive lenticule dissecting this plane, followed by the back surface of the lenticule. The lenticule was subsequently grasped with modified serrated McPherson forceps (Geuder, GmbH, Heidelberg, Germany) and removed. Then, the intrastromal space was flushed using a standard LASIK irrigating cannula. After surgery, steroidal medication (0.1% betamethasone, Rinderon; Shionogi, Osaka, Japan) and antibiotic medication (0.5% levofloxacin, Cravit; Santen) were topically administered 4 times daily for 2 weeks, after which the frequency was steadily reduced.

### Assessment of visual and refractive outcomes

We determined the logarithm of the minimum angle of resolution (logMAR) of uncorrected distance visual acuity (UDVA), the logMAR of corrected distance visual acuity (CDVA), and the manifest spherical equivalent or cylindrical refraction before and 3 months after surgery. Corneal astigmatism was measured using an autokeratometer (ARK-700A, Nidek Co. Ltd.).

### Vector analysis

Manifest refraction was converted to the corneal plane value before astigmatic vector analysis was done by Alpin’s method [11]. In both groups, the surgically induced astigmatism (SIA), target-induced astigmatism (TIA), astigmatic correction index, angle of error, difference vector, index of success, and flattening index were analyzed. The SIA is the vector magnitude of the actual change induced by surgery. The TIA is the vector magnitude of the intended change after surgery. The astigmatic correction index is the ratio of SIA to TIA. The angle of error is the difference between the angles of the SIA and TIA. The difference vector is the magnitude of astigmatic correction from the achieved result that is required to obtain the targeted goal. The index of success is calculated by dividing the difference vector by the TIA. An astigmatic correction index of 1.00 and an index of success of 0 indicate that the desired results have been obtained. The flattening effect is the amount of astigmatism reduction achieved by the effective proportion of the SIA at the intended meridian (flattening effect = SIA cos2×angle of error). The flattening index, which preferably equals 1, is obtained by dividing the flattening effect by the TIA. These vector analysis results were calculated and compared between the groups.

### Statistical analysis

All statistical analyses were performed using SPSS (SPSS Inc, Chicago, IL, USA). Sample size calculation was performed using PASS 2008 software (NCSS, Kaysville, Utah, USA). The Mann-Whitney *U* test was used to compare the visual acuity, the refraction, and the vector components between FLEx and SMILE and the Fisher’s exact test, to compare patient sex and the percentage of eyes within ± 0.50 D or ± 1.00 D of the manifest refractive cylinder correction between the two surgical techniques. The results are expressed as mean ± SD, and a value of p<0.05 was considered statistically significant.

## Results

### Patient population

Preoperative patient demographics are summarized in Table 1. All surgeries were uneventful and no definite intraoperative complications were observed. No epithelial ingrowth, diffuse lamellar keratitis, keratectasia or any other vision-threatening complications were seen at any time during the observation period in the FLEx or SMILE groups. No eye was lost during the 3-month follow-up in this series.

**Table 1:** Preoperative demographics of study population.

	FLEx group	SMILE group	p value	z value
No. of patients	26	26	-	-
No. of eyes	26	26	-	-
Age (years)	31.8 ± 6.1 (range, 21 to 44)	31.1 ± 7.1 (range, 20 to 45)	0.59	0.53
Gender (% female)	61.5	65.4	0.99	-
LogMAR UDVA	1.13 ± 0.15 (range, 0.82 to 1.52)	1.16 ± 0.25 (range, 0.40 to 1.52)	0.14	1.46
LogMAR CDVA	-0.21 ± 0.07 (range, -0.30 to -0.08)	-0.19 ± 0.08 (range, -0.30 to -0.08)	0.46	0.73
Manifest spherical equivalent (D)	-4.47 ± 1.43 (range, -7.00 to -2.50)	-4.87 ± 1.67 (range, -8.25 to -1.62)	0.31	1.04
Manifest cylinder (D)	-1.35 ± 0.57 (range, -1.00 to -2.75)	-1.37 ± 0.50 (range, -1.00 to -2.75)	0.55	0.33
Corneal astigmatism (D)	1.90 ± 0.84 (range, 1.00 to 3.75)	1.79 ± 0.86 (range, 0.00 to 3.75)	0.97	0.04

D = diopters; FLEx = femtosecond lenticule extraction; SMILE = small-incision lenticule extraction; logMAR = logarithm of the minimal angle of resolution; UDVA = uncorrected distance visual acuity; CDVA = corrected distance visual acuity.

### Visual acuity and refraction

**Fig 1:**
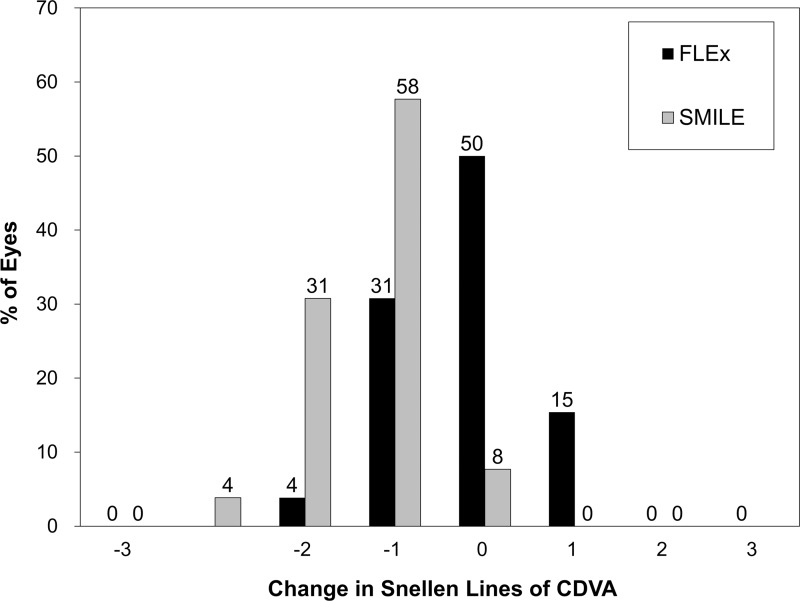
Changes in corrected distance visual acuity (CDVA) 3 months after femtosecond lenticule extraction (FLEx) and small-incision lenticule extraction (SMILE).

**Fig 2:**
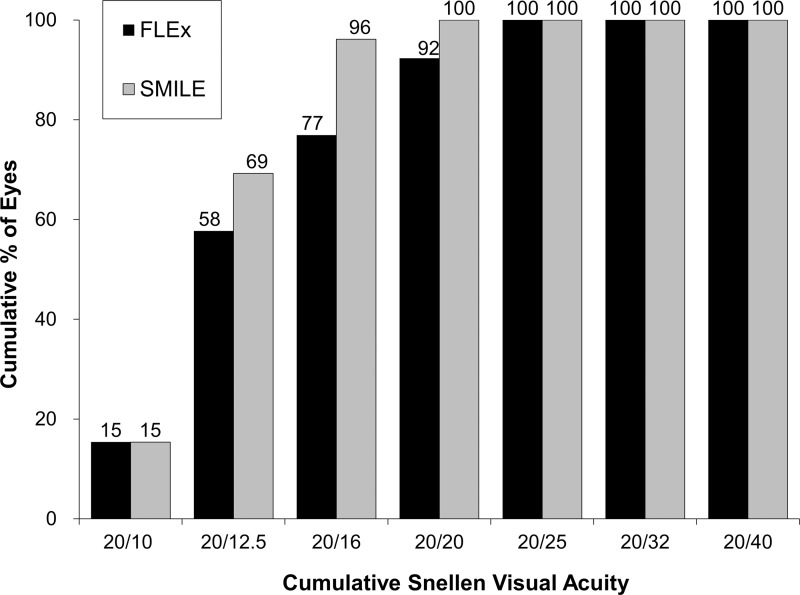
Cumulative percentages of eyes attaining specified cumulative levels of uncorrected distance visual acuity (UDVA) 3 months after femtosecond lenticule extraction (FLEx) and small-incision lenticule extraction (SMILE).

**Fig 3:**
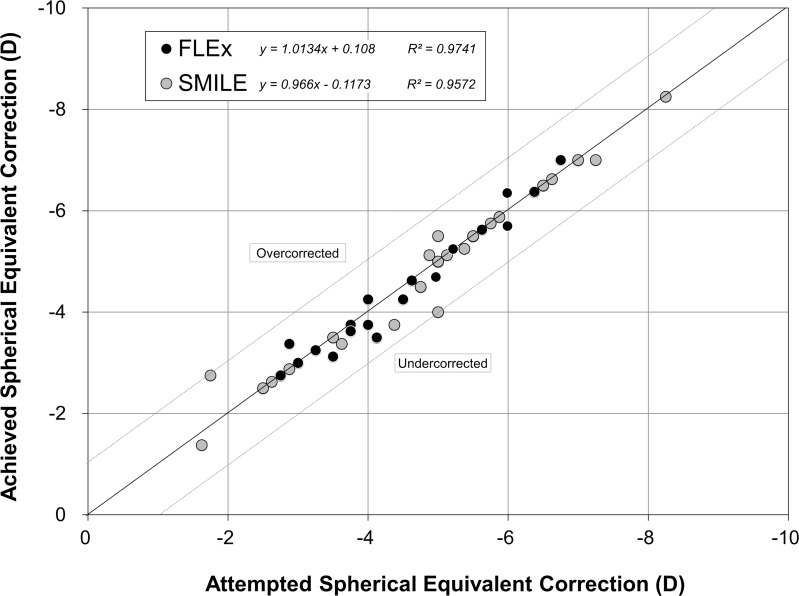
Ascatterplot of the attempted vs the achieved manifest spherical equivalent correction 3 months after femtosecond lenticule extraction (FLEx) and small-incision lenticule extraction (SMILE).

**Fig 4:**
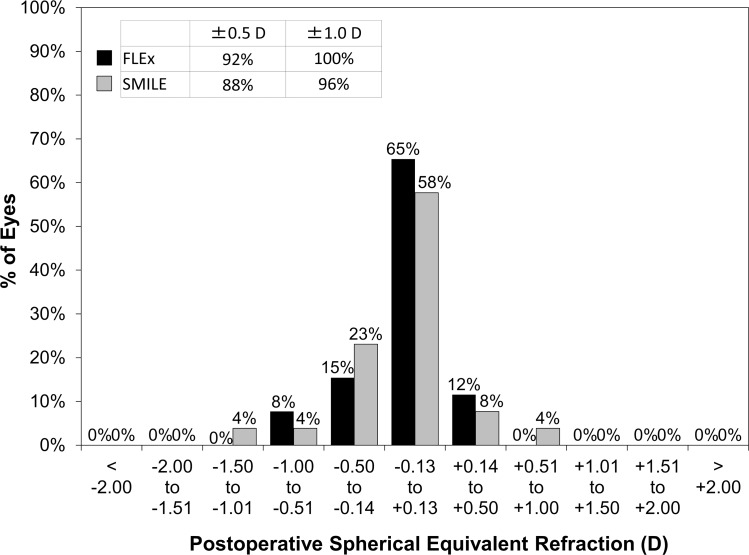
Percentages of eyes within different diopter ranges of the attempted correction (spherical equivalent) 3 months after femtosecond lenticule extraction (FLEx) and small-incision lenticule extraction (SMILE).

**Fig 5:**
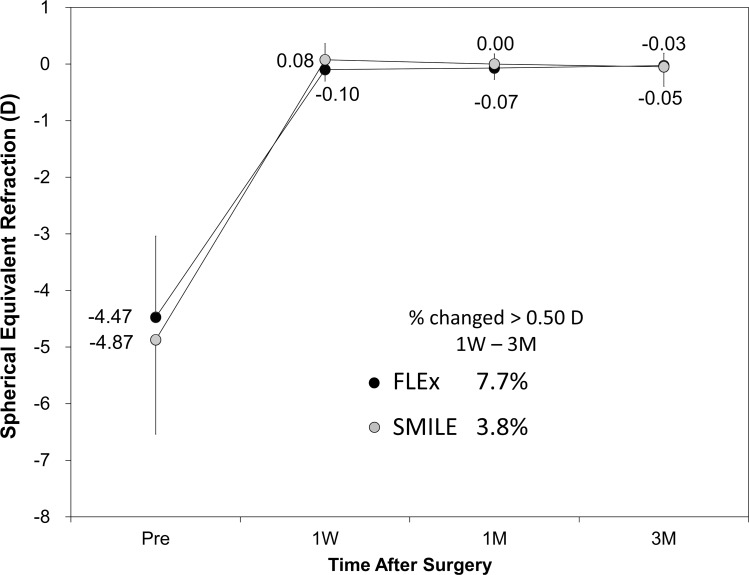
Time course of manifest spherical equivalent after femtosecond lenticule extraction (FLEx) and small-incision lenticule extraction (SMILE).

**Fig 6:**
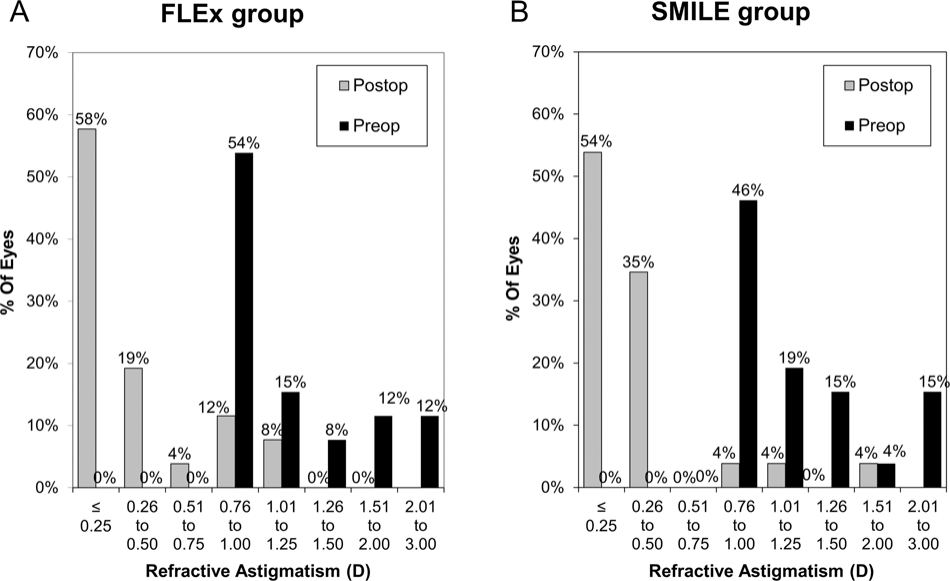
Percentage of eyes attaining specified levels of astigmatism before femtosecond lenticule extraction (FLEx) and small-incision lenticule extraction (SMILE) and 3 months after treatment.

The postoperative refractive and visual acuity outcomes at 3 months are shown in Table 2. There were no statistically significant differences in logMAR UDVA (p = 0.48, Mann-Whitney *U*-test), CDVA (p = 0.52), manifest spherical equivalent (p = 0.60), or manifest cylinder (p = 0.74) after surgery between the two groups. Fig 1 shows the percentage of eyes in which there was a gain or loss of Snellen visual acuity lines compared with preoperative levels at 1 week and 3 months. At 3 months postoperatively, 24 (92%) eyes in the FLEx group and 26 (100%) eyes in the SMILE group had UDVAs of 20/20 or better, respectively (Fig 2). Figs 3 and 4 show the difference in the achieved spherical equivalent correction versus the attempted correction. Fig 5 shows the stability and Fig 6, the refractive astigmatism.

**Table 2:** Postoperative visual and refractive outcomes in the femtosecond lenticule extraction (FLEx) group and the small-incision lenticule extraction (SMILE) group.

	FLEx group	SMILE group	p value	z value
LogMAR UDVA	-0.12 ± 0.11 (range, -0.30 to 0.05)	-0.09 ± 0.12 (range, -0.30 to 0.30)	0.48	0.71
LogMAR CDVA	-0.18 ± 0.07 (range, -0.30 to -0.08)	-0.16 ± 0.08 (range, -0.30 to 0.00)	0.52	0.64
Manifest spherical equivalent (D)	-0.03 ± 0.22 (range, -0.62 to 0.50)	-0.05 ± 0.34 (range, -1.00 to 1.00)	0.60	0.53
Manifest cylinder (D)	-0.38 ± 0.47 (range, -1.25 to 0.00)	-0.33 ± 0.45 (range, -1.75 to 0.00)	0.74	0.45
Corneal astigmatism (D)	1.00 ± 0.54 (range, 0.25 to 2.25)	1.31 ± 0.58 (range, 0.25 to 2.25)	0.50	1.95

D = diopters; FLEx = femtosecond lenticule extraction; SMILE = small-incision lenticule extraction; logMAR = logarithm of the minimal angle of resolution; UDVA = uncorrected distance visual acuity; CDVA = corrected distance visual acuity.

### Corneal astigmatism

There was also no statistically significant difference 3 months postoperatively in the corneal astigmatism between the two groups (p = 0.50).

### Refractive cylindrical correction

**Fig 7:**
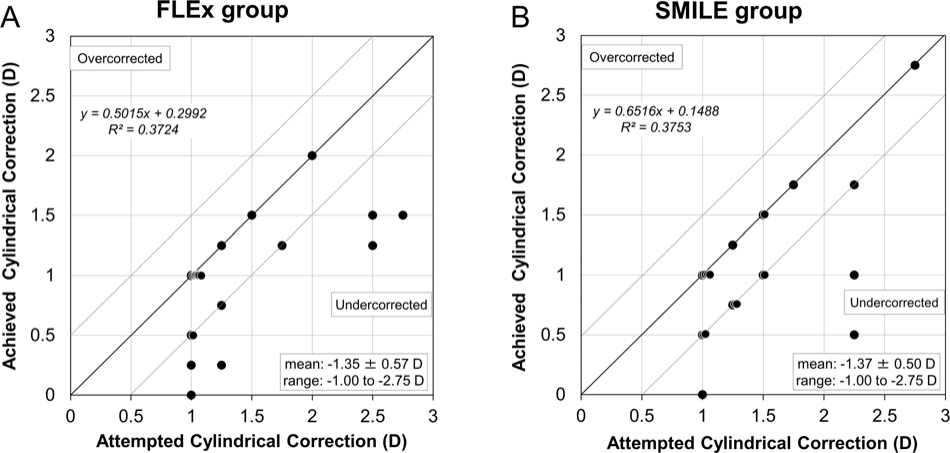
Ascatterplot of the attempted versus archived manifest cylindrical corrections 3 months after (A) femtosecond lenticule extraction (FLEx) and (B) small-incision lenticule extraction (SMILE).

The attempted versus the archived manifest cylindrical corrections made 3 months postoperatively, and the preoperative and postoperative manifest cylinder are shown in Fig 7. The predictabilities of manifest refractive cylinder corrections at 3 months are shown in Table 3.

**Table 3:** Predictability of manifest refractive cylinder correction in the femtosecond lenticule extraction (FLEx) group and the small-incision lenticule extraction (SMILE) group.

	FLEx group	SMILE group	p value
No. of eyes within ± 0.50 D (%)	20 (77)	23 (88)	0.47
No. of eyes within ± 1.00 D (%)	24 (92)	25 (96)	0.99

FLEx = femtosecond lenticule extraction; SMILE = small-incision lenticule extraction.

### Vector analysis

**Fig 8:**
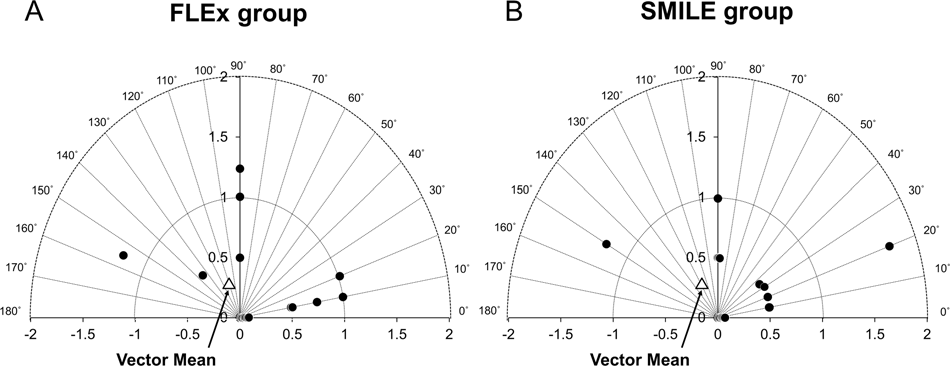
Vectorial display of the difference vector of manifest cylinder made 3 months postoperatively after (A) femtosecond lenticule extraction (FLEx) and after (B) small-incision lenticule extraction (SMILE).

The vector analysis results using 3-month refractive data are shown in Table 4. Alpin’s vector analysis showed that there were no significant differences in any astigmatic parameters (SIA, p = 0.80; TIA, p = 0.87; correction index, p = 0.77; angle of error, p = 0.24; difference vector, p = 0.76; index of success, p = 0.91; flattening effect, p = 0.79; flattening index, p = 0.84). Fig 8 shows the vectorial display of the difference vector of the manifest cylinder 3 months after surgery.

**Table 4:** Comparison of outcomes of the Alpins vector analysis in the femtosecond lenticule extraction (FLEx) group and the small-incision lenticule extraction (SMILE) group.

	FLEx group	SMILE group	p value	z value
SIA (D)	0.98 ± 0.36 (range, 0.33 to 1.92)	1.02 ± 0.48 (range, 0.20 to 2.56)	0.80	0.25
TIA (D)	1.20 ± 0.51 (range, 0.83 to 2.46)	1.20 ± 0.46 (range, 0.81 to 2.56)	0.87	0.17
Correction index	0.85 ± 0.24 (range, 0.36 to 1.22)	0.84 ± 0.22 (range, 0.22 to 1.00)	0.77	0.29
Angle of error (degrees)	0.40 ± 31.45 (range, -85.20 to 75.33)	-9.70 ± 33.71 (range, -86.92 to 71.27)	0.24	1.17
Difference vector				
Arithmetic (D)	0.37 ± 0.47 (range, 0.00 to 1.24)	0.33 ± 0.45 (range, 0.00 to 1.74)	0.76	0.31
Vector (D)	0.22 at 116°	0.22 at 120°	-	-
Index of success	0.28 ± 0.35 (range, 0.00 to 1.12)	0.26 ± 0.33 (range, 0.00 to 1.13)	0.91	0.11
Flattening effect	0.91 ± 0.39 (range, 0.05 to 1.69)	0.96 ± 0.48 (range, 0.10 to 2.56)	0.79	0.27
Flattening index	0.79 ± 0.30 (range, 0.05 to 1.10)	0.80 ± 0.26 (range, 0.11 to 1.00)	0.84	0.20

D = diopters; FLEx = femtosecond lenticule extraction; SMILE = small-incision lenticule extraction; SIA = surgically induced astigmatism; TIA = target induced astigmatism.

## Discussion

In the current study, we found no significant difference in the percentage of eyes within ± 0.50 D or ± 1.00 D between FLEx and SMILE techniques, indicating that both FLEx and SMILE are able to correct low to moderate astigmatism to similar degrees using Alpin’s method of vector analysis, and that the step of flap lifting in the FLEx procedure does not seem to induce astigmatism to the same significant extent as surgery does.

Although we present a relatively small sample size, this is to our knowledge the first published study comparing the astigmatic outcomes of FLEx and SMILE using Alpin’s vector analysis method. Every parameter used in this vector analysis has clinical relevance pertinent to the treatment of an eye after refractive surgery or to its outcome. This method has clinical benefits in the comparison of astigmatic correction between techniques, since it uses several parameters and provides a more realistic picture than simple numerical analysis of the treatment efficacy of a refractive procedure for astigmatism. There are a few studies available to validate the astigmatic correction of FLEx or SMILE [12–14]. When Ivarsen et al. [12] investigated the correction of myopic astigmatism using only the SMILE technique, they found significant undercorrection of astigmatism and increased errors in treatment resulting from attempts at higher degrees of correction. In the low-astigmatism group (<-2.50 D) included in their study, 77% of eyes were within ± 0.50 D and 95% were within ± 1.00 D of the intended correction of spherical equivalent 3 months postoperatively. Kunert et al. [13] reported that the index of success was 0.74 and the correction index was 1.20 at 3 months after FLEx, results which differed from ours in the FLEx group. The discrepancy may be attributed to differences in the distribution of preoperative astigmatism (-0.25 to -6.00 D vs. -1.00 to -2.75 D). Beyond the analysis of the two common parameters (index of success and correction index), the method of vector-based predictability analysis presented here allows quantification of the level of undercorrection or overcorrection of astigmatism. When this approach was used, our vector analysis results showed that the correction indices were less than 1.0, indicating an overall undercorrection of astigmatism, in both FLEx and SMILE groups. It is likely that, after gaining experience, a surgeon will be encouraged to correct the cylinders more accurately by using adjusted nomograms. We also demonstrated that the direction of an angle of error differed between FLEx and SMILE, although no significant difference was observed. Its positive value in FLEx showed that achieved correction is on an axis counterclockwise to its intended axis, whereas the achieved correction is clockwise to its intended axis in SMILE. The flattening index is a measure of the impact of an astigmatic treatment at off-axis orientation on the astigmatic change at its intended axis [11]. Insufficient flattening occurred in both FLEx and SMILE, as shown by a reduced median flattening index of 0.79 to 0.80.

It has been reported that the IntraLase flaps created with the femtosecond laser have better visual acuity and induce less astigmatism than the flaps created with the mechanical microkeratome in the LASIK procedure (0.22 D vs. 0.32 D), which indicates that the method of flap creation might dominate the surgically induced astigmatism, rather than the step of flap lifting [15–17]. Although the amount of astigmatism induced by making the corneal flap with the femtosecond laser is small, it is not clinically negligible for obtaining better visual performance after FLEx and SMILE techniques. In the present study, we found no significant difference in the surgically induced astigmatism between the FLEx and SMILE groups. Considering that SMILE is theoretically equivalent to FLEx as regards surgical technique except for the lifting of the flap, it is suggested that the presence or absence of flap lifting has little effect on the surgically induced astigmatism in ReLEx as an all-in-one device for lenticule processing. In the present study, we found no significant difference in the surgically induced astigmatism between FLEx and SMILE groups. Considering that SMILE is theoretically equivalent to FLEx for the surgical techniques except for the lifting of the flap, it is suggested that flap lifting, or its absence, has little effect on the surgically induced astigmatism in ReLEx as an all-in-one device for lenticule processing.

Possible reasons for the residual cylindrical errors observed in both FLEx and SMILE techniques could be cyclotorsion or astigmatic nomogram of the femtosecond laser system itself. Prakash et al. [18] reported that iris registration with eye tracking in LASIK gave better astigmatic results than when no iris registration was performed. In the current study, we performed both FLEx and SMILE without iris registration. We assume that, in both procedures, further improvements with compensation for cyclotorsion are required in order to achieve higher predictability for astigmatic correction, although the residual cylindrical errors in FLEx and SMILE were thought to be small, from a clinical viewpoint, at the 3-month follow-up.

There were at least two limitations to this study. Firstly, preoperatively, the study did not completely match the items in the patient backgrounds, such as age, gender, manifest spherical equivalent, and manifest astigmatism. These differences in the patient backgrounds may affect the astigmatic outcomes after ReLEx. Secondly, we determined the postoperative astigmatism 3 months postoperatively, when the corneal shape was considered to have been stabilized, taking into account the wound-healing responses of the cornea. A prospective randomized controlled study with a longer follow-up is necessary to confirm the authenticity of the results.

In conclusion, our results demonstrated that FLEx and SMILE were essentially equivalent in correcting astigmatism equal to or more than -1.00 D in terms of cylindrical refraction, the predictability of astigmatic correction, and astigmatic vector analysis components without vision-threatening complications occurring throughout the 3-month follow-up period. As far as we can judge, the presence or absence of flap lifting does not significantly affect the astigmatic outcomes after ReLEx. These novel surgical techniques may prove to be promising alternatives to excimer laser-based corneal refractive procedures for myopic astigmatism correction.
